# Allotrichophagia: A Unique Case of Parental Adjustment to Filial Pediatric Malignancy

**DOI:** 10.1155/2022/5949321

**Published:** 2022-06-15

**Authors:** Damir Huremović, Madhavi Latha Nagalla, Sameer Khan

**Affiliations:** ^1^North Shore University Hospital, A Division of Zucker-Hillside Department of Psychiatry, Northwell Health, Inc, USA; ^2^Donald and Barbara Zucker School of Medicine at Hofstra/Northwell, USA; ^3^Western Michigan University Homer Stryker School of Medicine, USA

## Abstract

A 36-year-old Hispanic female patient with gastrointestinal symptoms and weight loss was found to have a trichobezoar in her stomach requiring a surgical removal. Psychiatry team was consulted due to concerns for depression and trichotillomania. The psychiatric evaluation revealed that the patient was not ingesting her own hair - the most common instance in cases of trichotillomania and trichophagia, but her daughter's hair. The patient was doing this as an unconscious, spontaneous response to her daughter's manifest hair loss caused by daughter's malignancy and treatment thereof. The patient was given a diagnosis of Adjustment disorder and treated as such, as the patient's symptoms resolved with her daughter's remission. The patient's cultural background was taken into consideration and the team explored cultural factors that could have mediated such a response. The team also explored the psychodynamic aspects of this case in order to attain a more comprehensive understanding of this patient's unique presentation. To best describe this unusual behavior, we coined a term for such a phenomenon – **allotrichophagia** (Greek: eating others' hair).

## 1. Introduction

A 36-year-old Hispanic female patient with gastrointestinal symptoms and weight loss was found to have a trichobezoar in her stomach requiring a surgical removal [1]. Psychiatry team was consulted due to concerns for depression and trichotillomania [2]. In talking to the patient, the team understood that this finding was a consequence of patient's ingesting her daughter's hair as a response to her daughter's malignancy diagnosis and treatment [3–7]. A brief review of the literature yielded no cases of an individual ingesting *exclusively* another person's hair, which motivated us to report this case [8–10]. Given the patient's Hispanic background, we took into consideration the cultural factors that could have played a role in this response [11]. We also explored psychodynamic aspects of this case and were rewarded with a more complete understanding of this patient's unique presentation [12].

## 2. Case Presentation

Ms. Z was a 36-year-old Hispanic woman living with her partner and her two daughters. She had no significant psychiatric or substance abuse history. Ms. Z was Spanish-speaking only and interpreter services were used during all interactions with the patient. She presented to the emergency room with severe abdominal pain, nausea, vomiting, and constipation worsening over several weeks and was admitted to the general medicine floor for workup and management. During the workup, a large trichobezoar extending throughout her stomach and into her duodenum was identified as the cause of her symptoms. At that time, the primary team requested a psychiatry consult for trichotillomania and depression. During our evaluation and our interactions with her, Ms. Z was cooperative, soft-spoken and forthcoming with information. She was an immigrant from a Central-American country, with limited educational attainment and precarious legal status, working menial jobs to support her and her daughter. The patient was ambulatory, neatly dressed, overall pleasant but for the minimal anxiety related to her medical symptoms. Because of Ms. Z's limited command of English, the consultation process required a translator. Ms. Z's principal concern at the time of psychiatric consultation was the possible medical consequences of eating inedible materials.

On evaluation, Ms. Z endorsed feeling worried and depressed for several months in the year prior to this admission. One year earlier, her 16-year-old daughter was diagnosed with non-Hodgkin's lymphoma. Shortly after her daughter received the diagnosis, Ms. Z developed anxiety and became increasingly worried for her daughter's health. She experienced significant difficulty sleeping and had lost her appetite, losing 12 kg in the three-month period following her daughter's diagnosis [13]. Around that time, Ms. Z started ‘playing with her hair against the tongue' and recalled how the sensation of hair in her mouth had a comforting effect on her [12]. Ms. Z, however, emphatically denied pulling out and ingesting her own hair. She explained that, contemporaneously with playing with her hair on the tongue, her daughter was started on chemotherapy and started losing her own hair. Ms. Z recalled how difficult it was for her to see her daughter losing hair, as a dramatic evidence of her daughter's tribulations [14]. Ms. Z reported spending hours at her daughter's bedside, looking at the clumps of shed hair on the linen, and feeling a sudden and inexplicable craving to ‘eat that hair', a sensation she had never experienced before. Without thinking, she would start making a ball out of 4 or 5 strands of hair that she would then put in her mouth and chew for a long time. She recalled ‘loving the sensation of her daughter's hair against her tongue and melting away her anxiety' [12, 15]. After chewing hair for a long time, she would ultimately swallow the ball. Following this first ingestion, Ms. Z then carefully collected all her daughter's hair that was lost over one-week period in a plastic bag. For the next two months, Ms. Z would reach for the plastic bag whenever she found herself overwhelmed with anxiety and whenever she experienced the craving that would accompany her anxiety. She would pull some hair out from the bag and curl it into a small ball, then chew it ‘just like the bubble gum' for a while and then, after a while, she would swallow the ball. Ms. Z, however, did not engage in this activity every day and did not think much of it.

After two months, Ms. Z became ‘curious about her behavior' and researched it on the internet, as she wanted to know if her behavior was 'normal' and if not, if it would have any consequences for her health. She was terrified to learn that many people habitually engaged in hair pulling and ingestion and that it could lead to her ingesting other inedible things, such as mud or wall paint. At that point, she wanted to stop, but she still experienced the cravings she could not resist and continued to swallow her daughter's hair from time to time [16]. She also contacted her primary care doctor about her unusual behavior, who suggested that vitamin or other nutritional deficiency could also be implicated [17]. Ms. Z immediately started eating healthier and suppressed her cravings until she managed to completely stop eating her daughter's hair. Although she had stopped with this behavior, the craving still persisted as the uncertainty over her daughter's health lingered [16]. The following month, her daughter's health dramatically improved and the computed tomography scans showed that her daughter's tumor was shrinking in size. At the same time, the craving that Ms. Z had been experiencing for the past three months, disappeared.

Throughout this time, Ms. Z was not eating well and lost almost 12 kg during a three-month period. She also had difficulty sleeping and was intermittently tearful and sad, but denied any suicidality. Ms. Z denied any nightmares or flashbacks related to her daughter's treatment or any other traumatic experiences [3, 13].

Ms. Z was overall a healthy woman until her daughter's diagnosis, with no prior psychiatric issues in her childhood or throughout her adult life. Medically, Ms. Z was diagnosed with anemia when she was six years old. There was no history of substance abuse. Ms. Z was unsure about the family history of mental health and substance abuse. Ms. Z's medical family history included her daughter with non-Hodgkin lymphoma in remission.

## 3. Summary and Diagnosis

Ms. Z was diagnosed with Adjustment disorder with mixed disturbance of emotions and conduct after a careful deliberation on differential diagnosis that also included pica, obsessive-compulsive disorder proper, major depressive disorder single episode with/without psychotic features and trichotillomania (hair-pulling disorder) [18]. Initially, Ms. Z's symptoms pointed towards pica [19]. Her medical history of iron deficiency anemia and recent weight loss also pointed towards this diagnosis [20]. Pica is, however, statistically more common in children and in pregnant women and such behavior had not occurred in this 36-year-old mother of two children until her daughter started chemotherapy for non-Hodgkin's lymphoma [19].

Ms. Z's recurrent and persistent thoughts about her daughter's illness could be interpreted as obsessions and her act of hair ingestion as an anxiety-relieving compulsion. It is, however, difficult to understand Ms. Z's worry about daughter's health as excessive and unreasonable. In addition, Ms. Z's compulsions existed only in the context of an identifiable stressor and had neither preceded nor followed this circumscribed time period. Consequently, it would be inadequate to capture the totality of Ms. Z's symptoms as obsessive-compulsive disorder proper [18].

Another related diagnosis, from the same DSM-5 disorder spectrum (obsessive-compulsive and related disorders) - trichotillomania (and trichophagia), was also ruled out because Ms. Z had not been pulling out hair (absence of trichotillomania) and her hair ingestion (trichophagia) had been limited to her daughter's hair under very discrete, stressful circumstances [18]. In clinical practice, trichophagia is routinely described in individuals ingesting their own hair in the wake of trichotillomania [21]. Ms. Z, at the time of evaluation, did not present with any alopetic spots. More reliable evidence would have been collected by examining the proximal endings of individual hairs collected from her stomach via dermoscope that would distignush between pulled and spontaneously shed hairs [22]. This would have required a dermatology consult that had not been obtained at the time. In addition to having the identifiable stressor, however, Ms. Z also had other symptoms (sadness, tearfulness, anxiety) that could not be fully explained by the diagnosis of trichotillomania and trichophagia, but would suggest a diagnosis from mood spectrum disorder (depressive disorder) [18].

Ms. Z was sad, tearful, had trouble sleeping, lost significant weight, but she never had functional impairment, morbid preoccupation with worthlessness, suicidal ideation or psychomotor retardation [18]. Throughout her daughter's illness, Ms. Z always did what was expected of her as a mother and maintained her professional and familial functioning. Ms. Z never met full criteria for major depressive episode and her symptoms developed and persisted only in the context of a significant stressor; moreover, her symptoms abated once her stressful situation was resolved [18].

We then explored diagnostic entities associated with life events and traumatic stress. While it was conceivable that the daughter's experience was traumatic for Ms. Z, and she displayed almost dissociative quality to her symptoms, the duration of her symptoms and behaviors exceeded those of Acute stress disorder [3]. As time progressed, Ms. Z had not developed distinct clusters of symptoms required for a diagnosis of post-traumatic stress disorder (PTSD) [18]. We felt that the diagnosis of Adjustment disorder with mixed disturbance of emotions and conduct was the most appropriate [18]. It took into account the existence of an external stressor, its limited duration that coincided with the duration of her symptoms and accounted for both Ms. Z's emotional as well as behavioral symptoms. This unusual behavior was clearly related to the daughter's struggle with malignancy and had rapidly abated as the daughter's condition improved. Outside of this stressor, Ms. Z displayed no symptoms of impairment in her functioning and her symptoms were resolved shortly after the cessation of stress-inducing condition. A literature review yielded no reported cases of ingesting another person's hair without the presence of trichotillomania [8] and only sporadic cases of individuals with trichotillomania ingesting both their own and their mother's hair [9] or individuals ingesting artificial hair extensions [10]. To describe the unique phenomenon encountered in Ms. Z's case, we coined a term - ***allotrichophagia*** [Greek: eating others' hair].

## 4. Outcome

Patient was treated with exploratory laparotomy which showed a large trichobezoar filling the entire stomach cast with its tail extending into the duodenum. She was provided with supportive psychotherapy and started on mirtazapine, mainly to address her anxiety, sleep problems, and poor appetite, to which she responded well. She was referred for outpatient follow-up and continued to do well without recurrence of symptoms.

## 5. Cultural and Psychodynamic Formulation

Ms. Z was a single Hispanic mother with some immigration issues. Her coping abilities were evidently stretched thin under protracted stress. She was struggling to comfort herself when she was told about her daughter's life threatening medical condition [23]. Her behavioral patterns could appear as pathological emotional dysregulation if we were to disregard her cultural background. She comes from a more collectivistic background where people, when in distress, seek the support from their family, friends, religious leaders, or healers like “Curanderos” [24]. Pronounced reliance on support from family and community may find a person lacking individual coping skills when they find themselves in an unfamiliar environment. In this situation, the patient had no support from her family or the healers whom she has learned to rely upon when in distress [11]. The language barrier and her immigrant status further worsened the situation. Due to the language barrier, the patient did not comprehend well the various steps in the treatment and so was not well prepared for the physiological consequences of the treatment. Her uncertain immigrant status added to the financial burden and a sense of isolation [25]. Her 16-year-old English-speaking daughter was often the only connection between her and the foreign culture and at one point, there she was, standing alone, with no support, watching her daughter fade and the bridge to her new community collapse. She was overwhelmed by the fear of the loss of her loved one [26].

For Ms. Z, her daughter's hair can be interpreted as a totem or fetish, as evidenced by her vivid description of seeing it lying amidst her daughter's bedclothes and being attracted to it [12]. The totemic significance of hair is manifold. As a manifest part of her daughter's body, it was a vehicle for Ms. Z to feel closer to a love-object she feared she would lose. In olden times, it was common to give a loved one a lock of one's hair before a separation, to be cherished and worn around the neck [27]. Ms. Z went a step further and held the totem inside of her own body. Meanwhile the hair that was not immediately ingested was carefully collected and stored – to be savored later as sacrament or treasured as a memento. At the same time, the hair also represents death – hair itself is dead, and many cultures describe the tearing of hair as a mourning ritual [27]. By eating her daughter's hair, Ms. Z may have been trying to digest the incomprehensible facts of her daughter's illness and possible death.

The compulsion to eat the hair instead of simply storing it deserves further comment. Ms. Z herself associated her actions to ways she would self-soothe when she was younger and compared it to 'chewing bubblegum'. The sensory experience of feeling the hair in her mouth, accompanied by a sense of satiation, replicated the satisfaction of feeding in early life. Regression during chronic or life-threatening illness is commonly seen among patients and their families, and these aspects of Ms. Z's actions are evident. With orality comes the fantasy of being able to re-incorporate through ingestion, to forever make her daughter part of her own body, so as to never lose her. The satisfaction derived from eating the hair, however, was taboo, and lead to shame and guilt – leading the patient to scour the internet to find a disease label (complete with dire consequences) to censure herself with [28].

As her daughter's tumor continued to cause distress, Ms. Z had her own mass start to grow within her stomach. This may be interpreted as an unconscious attempt to identify with her daughter's disease, and join her in her suffering, or to replace the potential loss with a new “hair baby.” The latter may have led to further guilt, as may have unconscious fantasies about expelling her daughter from her body for good. This may have further reinforced the trichophagia as a punishment [28].

Utilizing a purely phenomenological, descriptive approach to psychiatric illness, based exclusively on symptom constellations may often overlook individuals' complexities and nuances. Seeking a more in-depth cultural and psychodynamic exploration and understanding makes this case stand out, whereas, approached merely phenomenologically, this case could have easily been misunderstood as a simple case of the obsessive-compulsive related disorder - trichotillomania.

## Data Availability

Access to data is restricted due to patient privacy.
